# Fidelity to an evidence-based model for crisis resolution teams: a cross-sectional multicentre study in Norway

**DOI:** 10.1186/s12888-021-03237-8

**Published:** 2021-05-04

**Authors:** N. Hasselberg, K. H. Holgersen, G. M. Uverud, J. Siqveland, B. Lloyd-Evans, S. Johnson, T. Ruud

**Affiliations:** 1 Division of Mental Health Services, Akershus University Hospital, Lørenskog, Norway; 2 Tiller Community Mental Health Centre, Department of Mental Health, St. Olavs Hospital, Trondheim, Norway; 3 University of South-Eastern Norway, Vestfold, Norway; 4 National Center for Suicide Research and Prevention, University of Oslo, Oslo, Norway; 5 Division of Psychiatry, University College London, London, UK; 6 Institute of Clinical Medicine, University of Oslo, Oslo, Norway

**Keywords:** Crisis resolution teams, Acute, Emergency, Implementation, Fidelity scale

## Abstract

**Background:**

Crisis resolution teams (CRTs) are specialized multidisciplinary teams intended to provide assessment and short-term outpatient or home treatment as an alternative to hospital admission for people experiencing a mental health crisis. In Norway, CRTs have been established within mental health services throughout the country, but their fidelity to an evidence-based model for CRTs has been unknown.

**Methods:**

We assessed fidelity to the evidence-based CRT model for 28 CRTs, using the CORE Crisis Resolution Team Fidelity Scale Version 2, a tool developed and first applied in the UK to measure adherence to a model of optimal CRT practice. The assessments were completed by evaluation teams based on written information, interviews, and review of patient records during a one-day visit with each CRT.

**Results:**

The fidelity scale was applicable for assessing fidelity of Norwegian CRTs to the CRT model. On a scale 1 to 5, the mean fidelity score was low (2.75) and with a moderate variation of fidelity across the teams. The CRTs had highest scores on the content and delivery of care subscale, and lowest on the location and timing of care subscale. Scores were high on items measuring comprehensive assessment, psychological interventions, visit length, service users’ choice of location, and of type of support. However, scores were low on opening hours, gatekeeping acute psychiatric beds, facilitating early hospital discharge, intensity of contact, providing medication, and providing practical support.

**Conclusions:**

The CORE CRT Fidelity Scale was applicable and relevant to assessment of Norwegian CRTs and may be used to guide further development in clinical practice and research. Lower fidelity and differences in fidelity patterns compared to the UK teams may indicate that Norwegian teams are more focused on early interventions to a broader patient group and less on avoiding acute inpatient admissions for patients with severe mental illness.

## Background

Crisis resolution teams (CRTs) are a part of the strategy in several high-income countries to reduce acute admissions to psychiatric wards by providing acute outpatient and home-based treatment. CRTs operating 24 h a day, 7 days a week are intended to offer immediate and intensive short-term emergency services in the community, be gatekeepers for admissions to acute wards, and facilitate early discharge from hospitals [1]. One earlier and two recent randomized controlled trials on CRTs showed that CRTs can reduce inpatient admissions, reduce bed use, and increase service users’ satisfaction with acute care [2–4]. However, there has been considerable variation in organisation and practice of CRTs, and there is limited knowledge of the implementation of the various components of the CRT model [5–9].

Three recent reviews have made more extensive overviews of the current knowledge of the effects of CRTs and related forms of crisis interventions for mental health crises. A Cochrane review of eight randomised clinical trials concluded that care based on crisis intervention principles appears to be a viable and acceptable treatment for people with serious mental illnesses [10]. Crisis intervention had been shown to reduce hospital admissions and family burden, and to improve mental state and global functioning. However, the evidence was of low to moderate quality. A systematic review of CRTs in adult mental health services included several kinds of studies [11]. Quantitative studies suggested that longer opening hours and the presence of a psychiatrist in the team may have increased the CRT’s ability to prevent hospital admission. Qualitative studies showed that stakeholders emphasized communication and collaboration, treatment at home, and limiting the number of team members meeting the service user. A rapid synthesis of the evidence for outcomes of available models of mental health crisis care included one review of reviews, six systematic reviews, nine guidelines and 15 primary studies [12]. There was positive evidence for the clinical effectiveness of CRTs but variability in implementation, and the evidence was of low quality. The authors of the two last reviews emphasised the need for a more clearly defined CRT model, as well as for a fidelity scale which can measure the components of CRT and improve the quality of research on the model and its key components.

Fidelity is defined as the degree to which a program implementing an evidence-based practice (EBP) adheres to specific model standards, which have been documented to give positive clinical outcome [13]. Fidelity scales are tools to measure the degree of implementation of an EBP and its key components. Such scales may be used to guide and monitor implementation of an EBP, compare implementation across sites, support sustainability of an EBP, document the association between outcomes and implementation of the EBP, and identify the importance of various components of an EBP. The development of fidelity scales has been a natural outgrowth of the increased emphasis on EBPs [14].

The CORE Crisis Resolution Team Fidelity Scale was developed by the CORE (Crisis resolution team Optimization and RElapse prevention) study and is the first fidelity scale measuring the CRT model [15]. The fidelity scale was developed through a comprehensive process, integrating a review of existing literature and qualitative interviews with various stakeholder groups to identify CRT core elements, followed by a concept mapping procedure to prioritize and group these elements, and finally operationalisation and calibration of the elements into specific fidelity scale items [15]. The fidelity scale was pilot tested on 75 CRTs in the UK [9, 15] and on 24 CRTs in Norway. Results from using the fidelity scale have been published from a survey and cluster-randomised trial in the UK and in a report on preliminary results from the current study in Norway [3, 9, 16]. The UK cluster randomised trial provided some preliminary validation of the scale, as higher fidelity scores were associated with fewer admissions and less inpatient bed use, though not clearly greater patient satisfaction for CRT service users [3].

There is a lack of knowledge on the implementation of the CRT model outside the UK**,** and the fidelity scale may be used to compare CRT implementation across countries. Norway is the only country outside the UK where CRTs have been designated as mandatory within mental health services. This was done by Norwegian health authorities in 2005, inspired by the implementation of CRTs in the UK in 2000. However, a study in 2005 found that none of the first eight Norwegian CRTs operated 24 h a day, 7 days a week, and none had gatekeeping functions for acute wards [5]. The CRTs also treated patients who were not considered for hospital admission. A survey of the 56 CRTs in Norway in 2015 indicated large variations in practice, but it did not measure how the teams’ practices were compared with the evidence-based CRT model or with the national recommendations for CRTs published by the Norwegian Directorate of Health [17, 18].

Norway has 5.4 million inhabitants and a geography with low population density and long distances to services for many people. Specialized mental health services are run by 19 health trusts owned by four regional health authorities on behalf of the state. They include 66 community mental health centres (CMHCs) and acute psychiatric hospital wards [19]. The CMHCs provide local inpatient services as well as outpatient services, including some mobile services by CRTs, teams for early intervention in psychosis, assertive community treatment (ACT) teams, or functional assertive community treatment (FACT) teams [20]. Due to variation in resources and geographics there are some variations in capacity and available services, but outpatient and mobile team services are available both in urban and rural areas. Fifty-six of the CMHCs had a CRT in 2014 [18].

### Aims

The aims of the study were to explore whether the CORE Crisis Resolution Fidelity Scale was applicable and relevant to measuring CRT model fidelity in Norway and to examine the fidelity of Norwegian CRTs to an evidence-based CRT model. To our knowledge, this is the first study which has used the fidelity scale to assess CRTs outside the UK.

## Methods

### Study design

This fidelity study was a part of a multicentre study of CRT treatment outcomes [16]. The study was planned and conducted in collaboration with a national network of acute mental health services providers, and most of the CRTs in the country participated in this network.

### Sample

The 28 CRTs were from rural and urban areas in all four health regions, and they were considered representative of the 56 CRTs in the country. The CRTs signed up to participate in the multicentre study in response to an invitation sent to mental health services in all the health trusts in Norway. There were no exclusion criteria for participation in the study. The catchment areas for the 28 CRTs had a population from 40,000 to 130,000. The CRTs were from 15 of the 19 health trusts in Norway and from all parts of the country. Nine teams were in the three major university cities, and most of the others were in towns serving also surrounding rural areas. All the CRTs were located close to local bed units of the CMHC. Very few teams were located together with acute psychiatric hospital wards, but half of the teams were in cities or towns with an acute psychiatric hospital department. CRTs in smaller CMHCs and in Northern Norway are underrepresented in the study.

### Measure

We measured CRT model fidelity using the CORE Crisis Resolution Team Fidelity Scale Version 2 with 39 items [15]. The scale was developed in the UK based on evidence-based practice principles to capture stakeholder views of what constitutes best practices in CRTs. Version 1 of the fidelity scale was piloted in the UK and Norway, and the piloting led to some adjustments for version 2 of the scale. Items are scored on a scale of 1–5, where 4 and 5 are considered high fidelity, 3 is considered moderate fidelity, and below 3 is considered low fidelity. The fidelity scale consists of four subscales measuring referrals and accessibility (10 items), content and delivery of care (16 items), staffing and team organisation (10 items), and location and timing of care (3 items). All material was translated from English into Norwegian. In the UK, interrater reliability has been tested by asking 16 fidelity assessors to score an extended case note vignette of a CRT. The mean estimated correlation between individual item ratings was 0.65 (CI: 0.54 to 0.76), and the estimated intraclass correlation (ICC) between assessors was 0.97 for the total fidelity score [15].

### Data collection and procedures

The fidelity assessments were completed between April and June 2015 by seven trained fidelity assessors. The evaluation team visiting each CRT consisted of three members: two with work experience on a CRT and one with mental health service user experience. Four of the assessors had participated in the pilot of version 1 of the fidelity scale in 2014. The evaluation team leader had extensive CRT experience as a CRT manager and clinician, from her PhD study on the first eight CRTs in Norway, and as evaluation team leader for piloting version 1 of the fidelity scale in Norway.

The evaluation team visited each CRT for 1 day. The team read case notes and procedures, brochures, and statistics of the CRT. Interviews were conducted with the team manager, team members, and key informants from collaborating agencies such as acute wards and primary care. Case notes were reviewed for the last ten service users that the CRTs had discharged. Structured face to face or telephone interviews with six recent service users and six carers (family members) were conducted by the assessor with service user experience. Interrater reliability was not tested in our study. However, we aimed to avoid inconsistency of ratings across CRTs by having consensus rating by the three evaluation team members visiting a CRT, and by variation in which three of the seven evaluation team members assessed a CRT.

At the end of the visit, the evaluation team gave the CRT preliminary feedback on the key points in their assessment. After a few weeks, the CRT received their fidelity scores and a draft with written comments accompanying the scores. The CRT could comment on the scores and provide any information not available during the visit. The evaluation team then finalised the fidelity scores and sent these to the CRT. The evaluation team coordinator was available for questions from the CRTs through the entire evaluation process. After finishing the fidelity assessment of all the CRTs, the fidelity assessors and the principal investigator held a meeting summarizing the experiences of conducting the fidelity assessments and discussing whether the fidelity scale was applicable to assess the Norwegian CRTs.

In March 2017, the principal investigator conducted a brief semi-structured telephone interview with 16 CRT managers who responded to a request for such an interview. The interview was about the experiences of participating in the study and any actions taken based on those experiences and on the feedback from the fidelity assessment or from tables on team and patients characteristics (see below).

### Data analysis and reporting

Data was analysed with descriptive statistics using the statistical software SPSS for Windows version 23. Distribution of scores and median scores were reported for each item. Descriptive statistics (mean, SD) and distribution of mean scores were reported for the four subscales and for total fidelity. The applicability of using the fidelity scale to assess Norwegian CRTs was briefly summarized based on the comments from the fidelity assessors and the CRT managers.

The results from the multi-centre study, including the results from the fidelity assessment, were presented to the CRTs in a meeting in the national network for acute mental health services in April 2016, as well as to national health authorities and CMHC directors in their annual conference in May 2016. In September 2016, the principal investigator of the multi-centre study also gave each CRT feedback with detailed tables on characteristics of the team’s patients, practice, and outcome from the data recorded by the team during the outcome study.

## Results

### The applicability of the fidelity scale to Norwegian crisis resolution teams

To our knowledge, this is the first study outside the UK reporting the fidelity of CRTs. All of the items in the CORE Crisis Resolution Team Fidelity Scale could be rated for all participating CRTs. In the meeting summarizing the experiences of the fidelity assessments, the fidelity assessors agreed that the fidelity scale was applicable for assessment of Norwegian CRTs. In the semi-structured telephone interviews, the CRT managers expressed that the feedback from the fidelity assessment and the clinical results had been used by the teams in discussions and reflections on their clinical practice, as well as in decisions on further developments of the CRTs.

### The model fidelity of the crisis resolution teams

Table 1 shows the distribution and median fidelity score for each item. The median fidelity rating was 1–2 (low) for 18 items, 3 (moderate) for 8 items, and 4–5 (high) for 13 items. Median fidelity was high for accessibility for referrals, having a psychiatrist on the team, comprehensive assessments, providing individualized care, providing psychological interventions, visit length, consistency of care, service user’s choice regarding location and types of support, and consistency of staff and support during the care.

**Table 1:** Distribution and median of fidelity scores on items for 28 Norwegian crisis resolution teams

Subscales and items	Fidelity					
	1	2	3	4	5	Median
**1.Referrals and accessibility**						
1.The CRTs responds quickly to new referrals	9	4	7	5	3	3
2.The CRT is easily accessible to all eligible referrers	1	0	2	8	17	5
3.The CRT accepts referrals from all sources	2	2	5	2	17	5
4.The CRT will consider working with anyone who would otherwise be admitted to adult acute psychiatric hospital	0	0	0	7	21	5
5.The CRT provides a 24-h, seven days a week service	28	0	0	0	0	1
6.The CRT has a fully impended “gatekeeping” role, assessing all patients before admission to acute psychiatric wards and deciding whether they are suitable for home treatment	26	2	0	0	0	1
7.The CRT facilitates early discharge from hospital	28	0	0	0	0	1
8.The CRT provides explanation and direction to other services for service users, carers and referrers regarding referrals which are not accepted	0	1	5	17	5	4
9.The CRT responds to requests for help from service users and carers whom the CRT is currently supporting	2	2	1	18	5	4
10.The CRT is a distinct service which only provides crisis assessment and brief home treatment	10	7	7	4	0	2
**2.Content and delivery of Care**						
11.The CRT conducts a comprehensive assessment for all service users accepted for CRT support	2	4	7	1	14	4
12.The CRT provides clear information to service users and families about treatment and visits	0	1	21	5	1	3
13.The CRT closely involves and works with families and wider social networks in supporting service users	11	7	5	3	2	2
14.The CRT assesses carers’ needs and offers carers emotional and practical support	0	23	4	1	0	2
15.The CRT reviews, prescribes and delivers medication for all service users when needed	17	7	4	0	0	1
16.The CRT promotes service user’ and carers’ understanding of illness and medication and addresses concerns or problems with medication	8	16	3	0	1	2
17.The CRT provides psychological interventions	0	1	2	17	8	4
18.The CRT considers and addresses service users’ physical health needs	5	5	5	11	2	3
19.The CRT helps service users with social and practical problems	0	1	14	5	8	3
20.The CRT provides individualized care	1	2	3	8	14	4
21.CRT staff visits are long enough to discuss service users’ and families’ concerns	0	1	8	12	7	4
22.The CRT prioritises good therapeutic relationships between staff and service users and carers	0	3	25	0	0	3
23.The CRT offers service users choice regarding location, timing and types of support	1	1	6	17	3	4
24.The CRT helps plan service users’ and service responses to future crises	27	0	0	0	1	1
25.The CRT plans aftercare with all service users	3	22	3	0	0	2
26.The CRT prioritises acceptability to service users in how CRT care is ended	2	7	3	9	7	4
**3.Staffing and team organisation**						
27.The CRT has adequate staffing levels	15	2	3	3	5	1
28.The CRT has a psychiatrist or psychiatrists in the CRT team, with adequate staffing levels	3	9	1	0	15	5
29.The CRT is a full multi-disciplinary staff team	7	5	9	6	1	3
30.The CRT provides a thorough introduction program for new staff and ongoing training and supervision in core competencies for CRT staff	21	4	2	1	0	1
31.The CRT has comprehensive risk assessment and risk management procedures, including procedures for safeguarding children and vulnerable adults living with CRT service users	14	8	5	1	0	1
32.The CRT has systems to ensure the safety of CRT staff members	2	13	4	5	4	2
33.The CRT has effective record keeping and communication procedures to promote teamwork and information sharing between CRT staff	2	11	6	7	2	3
34.The CRT works effectively with other community services	5	13	8	1	1	2
35The CRT takes account of equality and diversity in all aspects of service provision	0	5	21	2	0	3
36.The CRT has systems to provide consistency of staff and support to a service user during a period of CRT care	0	4	6	5	13	4
**4.Location and timing of care**						
37.The CRT can access a range of crisis services to help provide an alternative to hospital admission for service users experiencing mental health crisis	8	9	10	1	0	2
38.The CRT provides frequent visits to service users	27	1	0	0	0	1
39.The CRT mostly assesses and supports service users in their home	17	7	1	0	3	1

Median fidelity was low for opening hours, adequate staffing levels, gatekeeping acute psychiatric inpatient admissions, facilitating of early discharge from hospitals, providing medication, plans for response to future crisis, introduction program for new staff and ongoing supervision, risk assessment and risk management, home treatment and intensity of care, family involvement, and collaboration with other services.

**Fig. 1:**
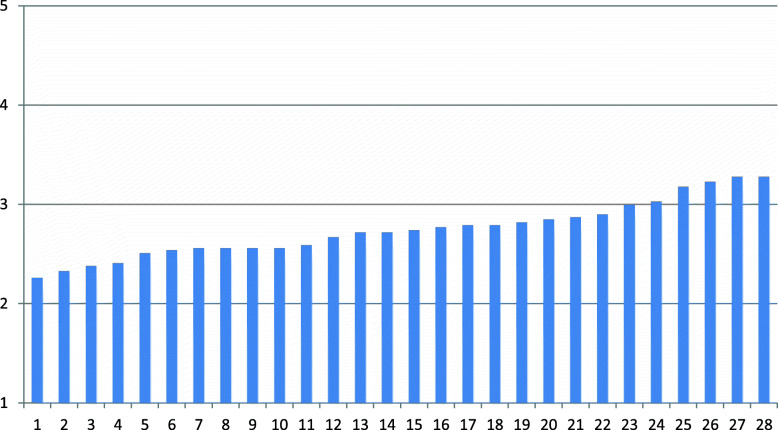
Mean fidelity score of 28 crisis resolution teams ordered with increasing score

Table 2 shows that the mean fidelity item score for the 28 CRTs was 2.75 (SD 0.28) (low fidelity). The mean fidelity score ranged from 2.26 to 3.28 (low to moderate fidelity), with a median of 2.74 (low fidelity). There was a moderate variation in mean fidelity among the CRTs. The content and delivery of care subscale showed the highest fidelity score, and the location and timing of care subscale showed the lowest score. No team had a mean fidelity item score of 4 or higher (high fidelity), and 6 teams (21%) had a mean item fidelity score between 3 and 4 (moderate fidelity). Figure 1 shows the mean total fidelity score for each of the 28 crisis resolution teams, which is within the low to moderate fidelity range.

**Table 2:** Mean fidelity for subscales and mean total fidelity for 28 Norwegian crisis resolution teams

			Distribution of fidelity				
Subscales	Items	Mean (SD)	1.00–1.99	2.00–2.99	3.00–3.99	4.00–5.00	Median
1.Referrals and accessibility	10	2.88 (0.40)	0	13	15	0	3.00
2.Content and delivery of care	16	2.93 (0.44)	0	13	15	0	3.03
3.Staffing and team organisation	10	2.65 (0.47)	2	20	6	0	2.55
4.Location and timing of care	3	1.64 (0.59)	21	5	2	0	1.67
**Total fidelity**	**39**	**2.75 (0.28)**	**0**	**22**	**6**	**0**	**2.73**

## Discussion

The fidelity assessment using the CORE Crisis Resolution Team Fidelity Scale was experienced as applicable by the fidelity assessors and as useful by the CRT managers in this first published report using the fidelity scale outside the UK. The Norwegian CRTs had low to moderate fidelity scores, with a moderate variation among the teams. The highest fidelity scores were on the content and delivery of care subscale, and lowest were on the location and timing of care subscale.

Mean fidelity was lower in Norway than in the UK, and only 21% of the Norwegian teams achieved moderate fidelity (score of 3 or more) compared to 57–65% of the UK teams [3, 9]. This raises the question as to whether the Norwegian teams were trying to implement a somewhat different model of CRT, or whether they simply had a less robust implementation of the model. Below we comment on the results in our study for various aspects in each of the four subscales, and we also compare them to results from UK studies [3, 7, 9, 15]. As we have not found any published results using the fidelity scale from any other country, we are only able to compare our results to results from the UK.

### Referrals and accessibility

Various Norwegian agencies can refer patients to CRTs, similar to the UK. There was a large variation among the CRTs in response time to new referrals, which was comparable to response time of UK teams, ranging from 1 h to 1 week [7]. Most CRTs were a part of the CMHC outpatient services and were often given the task of assessing emergency referrals on behalf of the CMHC outpatient clinic. Thus, many CRTs had to do such assessments for the outpatient clinic in addition to working with patients who would otherwise be admitted to acute psychiatric wards.

No Norwegian teams operated 24/7. However, 16 teams (57%) operated extended hours on weekdays, and six of these also operated some hours on weekends. The remaining 12 teams (43%) operated only during office hours on weekdays. In UK 70% of teams operated 24/7 [7]. In the UK, 24/7 availability was an explicit goal in the National Health Services Plan of 2000 [21], while in Norway 24/7 availability was considered by the health trusts to demand too many resources for small catchment areas. It was expected that most needs during nights would be met by GPs on call and other primary health services available 24/7. Still, 57% of Norwegian CRTs had extended opening hours, but this did not impact their fidelity ratings, which required 24/7 to achieve a high accessibility rating on this item. According to a Norwegian study and a systematic review, extending opening hours may contribute to prevent hospital admissions [11, 22].

None of the CRTs had a gatekeeping role for acute admissions to acute psychiatric wards, in contrast to most UK teams. This also means that patients brought by the police to involuntary admissions in acute psychiatric departments usually did not come to the CRT. This is probably different from the UK, where most CRTs are gatekeepers of acute psychiatric beds and where street triage involves the police [23]. The CRTs also did not facilitate early discharge from hospitals, while many UK teams did. Several Norwegian CRTs advocating low thresholds and early interventions have argued that many people in crisis may need a CRT with a “gate opening” role to a short inpatient stay, rather than a gatekeeping role delaying admission to later when the crisis has become more serious. The recommendations from the Norwegian health authorities and the decisions of the health trusts have not given CRTs responsibility for gatekeeping admissions to acute psychiatric wards, even if they are expected to contribute to reducing acute admissions. With a higher number of psychiatric beds per 100,000 inhabitants in Norway than in the UK [24], the threshold for acute admissions is probably lower in Norway, and with less pressure to avoid admissions.

### Content and delivery of care

Half of the CRTs were providing a comprehensive assessment, which was higher than in the UK. One possible reason may be a need for broader assessments to cover the wider range of patient groups accepted by the Norwegian teams [5]. Norwegian teams were rated high on giving psychological interventions and low on giving medication, which is the opposite of UK teams. This may indicate differences in patient groups served, composition of teams, or team procedures. UK teams have a larger proportion of service users with severe mental illness compared to the Norwegian teams [5]. Norwegian teams had high scores for emphasis on services users and carers, but low on family involvement (as the teams supported families mainly if contact was initiated by the family). This finding was surprising, because there has been enthusiasm in many Norwegian CRTs for an open dialogue approach involving family and network [25]. Both Norwegian and UK teams had moderate scores regarding giving physical health care and emotional support, and low on plans for early identification and intervention in future crises.

### Staffing and team organisation

Norwegian CRTs are multidisciplinary with mental health nurses as the largest professional group, and most teams had a psychiatrist at least part time, as well as a clinical psychologist. A systematic review found that the presence of a psychiatrist in the team may contribute to prevent hospital admissions [11]. However, the general level of staffing was rated as moderate for more than half of the teams, and training and supervision of team members was rated low. There was a variation in systematic procedures to ensure safety for team members, and the risk assessment for service users and carers was rated low. Communication and information sharing between team members and consistency of support to service users were moderate to high, and collaboration with other community services was low to moderate. Most UK teams were rated as having high staffing and a medium level of training and supervision.

### Location and timing of care

Norwegian teams only partly provided home-based care, compared to UK teams, which usually provided home-based care. Norway is a country with scarce population density in most areas, making delivery of home treatment time consuming due to long travel distances. Norwegian teams were found to deliver less intensive care regarding frequency of visits, while the length of visits was high compared to UK. Qualitative studies have shown that service users of CRTs appreciate that team members have time for listening and communication [11]. High emphasis on psychological help and long distances travelled for many teams may be factors explaining some of these differences.

#### Factors influencing the Norwegian crisis resolution teams

Several factors may have contributed to the practices of the Norwegian CRTs and to differences compared to UK CRTs. The national recommendations for CRTs are more specific in the UK than in Norway, where there are fewer restrictions on target groups and less emphasis on gatekeeping functions of acute psychiatric beds [17, 21]. This may indicate that higher priority of CRTs’ role in mental health policies and health trusts are needed to implement an evidence-based CRT model with extended opening hours, gatekeeping functions, facilitation of early discharge from hospital, and more intensive treatment with frequent visits to service users’ homes.

The CRTs in Norway were influenced by two visions which may have led to conflicting or different priorities and practices. Establishing CRTs throughout Norway was based on national plans and experiences regarding CRTs in the UK, and some of the pioneering CRTs in Norway had UK CRT experts as supervisors and/or visited UK CRTs. On the other side, the growing emphasis on early intervention and on low threshold services also inspired many Norwegian CRTs. Thus, teams in Norway have partly aimed for lower thresholds and earlier interventions in crises and a broader target group, and partly adapted the CRT model to more rural areas with longer distances to travel for staff and service users. The focus may have been more on the content with more psychological and less medical treatment for a broader patient group than on avoiding acute admissions for patients with the most serious illnesses.

Although some CRTs scored high fidelity on several items, no teams scored consistent high fidelity across all items or achieved optimal overall fidelity. This was the same in the UK, even though overall fidelity was higher there. This may reflect that a CRT is a complex intervention with many elements. It is hard to practice all components well, and sometimes the components can compete with each other. For instance, if teams prioritise easy access and rapid response to new referrals, they have less time to provide frequent visits and intensive home treatment to service users–and vice versa. This challenge may be a driver for the observed trend in England of CRTs splitting into two separate services for crisis assessment and crisis home treatment [7].

Findings in the CORE trial and a review indicate that different CRT components may be critical for different outcomes [3, 11]. Extended opening hours seem to be a critical component for reducing admissions [11, 22], and focus on early discharge has also been shown to reduce hospital use [4]. As the Norwegian CRTs did not score highly on either of these functions, they may have limited effect on use of inpatient services. Content of care matters for patient satisfaction [3], and the emphasis in Norwegian CRTs on psychological treatment and longer meetings may be associated with higher patient satisfaction. Studying how fidelity scores relate to patient outcomes and experiences will be important steps in further research using the CRT Fidelity Scale.

The fidelity scale was based on the best available evidence for what is considered core components of crisis resolution teams. However, in operationalising the rules for rating each component, decisions were made on the criteria which needed to be met for each level of fidelity. For some items, the criteria may need to be changed to avoid floor or ceiling effects. The extended opening hours of 57% of the Norwegian CRTs were not reflected in their fidelity score, even if such extended opening hours have been shown to be associated with reduced risk for acute inpatient admissions [22]. Eventual recalibration of items needs more data and experience, as well as opportunities to test revised versions of the fidelity scale [14].

The fidelity assessors found the fidelity scale to be applicable for measuring Norwegian CRTs, and the CRT managers found the feedback from the fidelity review useful for further development of their teams. However, parts of the fidelity scale may discriminate against CRTs aiming to provide early interventions with low thresholds for a broader range of patients, compared to serving as an alternative for seriously ill patients who would otherwise need acute inpatient admission. The two attempts at a nationwide implementation in the UK and Norway seem to have resulted in substantially different versions of the CRT model, and likely with different outcomes.

#### Strengths and limitations

The study used a comprehensive fidelity scale developed by a leading research group on crisis resolution teams, and several of the Norwegian evaluation team members had experience from the piloting of the fidelity scale. One limitation was that the 28 CRTs in this study signed up voluntary to participate, so the sample may have been biased with overrepresentation of teams with more engagement in getting feedback on their practice. CRTs in smaller CMHCs and in Northern Norway are underrepresented in the study. Another limitation was that we could not calculate interrater reliability of the fidelity ratings, because the members of the evaluation teams did not do independent ratings of fidelity before agreeing on scores by consensus.

## Conclusions

The CORE CRT Fidelity Scales are a valuable tool to measure CRT practice for local feedback and team development, and to research which components are critical for which outcomes. Lower fidelity and differences in fidelity patterns compared to the UK teams may indicate that Norwegian teams have more focus on early interventions to a broader patient group and less on avoiding acute inpatient admissions for patients with severe mental illness.

## Data Availability

The datasets used and/or analysed during the current study are available from the corresponding author on reasonable request.

## Abbreviations

CRT: Crisis resolution team
CMHC: Community mental health centre
EBP: Evidence-based practice
